# Fascin is secreted in male's serum: results of a pilot study

**DOI:** 10.4155/fsoa-2017-0098

**Published:** 2018-01-05

**Authors:** Daniel Porav-Hodade, Orsolya Martha, Daniel Balan, Sabin Tataru, Adina Hutanu, Anca Sin, Mihai Dorin Vartolomei

**Affiliations:** 1 University of Medicine & Pharmacy Tirgu Mures, Department of Urology, Tirgu Mures, Romania; 2 University of Medicine & Pharmacy Tirgu Mures, Department of Cell & Molecular Biology, Tirgu Mures, Romania; 3 University of Medicine & Pharmacy Tirgu Mures, Department of Laboratory Medicine, Tirgu Mures, Romania; 4 Medical University Vienna, Department of Urology, Vienna, Austria

**Keywords:** biomarker, fascin, serum

## Abstract

**Aim:**

Fascin is a 55 kDa globular protein with an important role in cell migration. Aim of study was to investigate serum fascin in healthy males.

**Materials & methods:**

From 1 July 2016 to 31 December 2016, we collected serum from 46 males. Serum fascin level was performed using ELISA kit from USBiological (Salem, MA, USA).

**Results:**

Median age was 64 years. Mean fascin serum level was 9.84 ng/ml, mean prostate-specific antigen (PSA) was 2.74 ng/ml and mean prostate volume was 37.64 cc. The 51–60 years group had a mean of 10.53 ng/ml, the 61–70 group a mean of 9.7 ng/ml and the 71–80 group had a mean of 9.41 ng/ml fascin serum level.

**Conclusion:**

Fascin serum level did not differ according to age in males.

## Lay abstract

Fascin protein was previously associated with high-grade malignancies. This study presents the determination of fascin serum levels in 46 healthy males, providing a reference number in further determinations. The patients were divided into three subgroups according to age. The study presents the mean levels of each group, in association with PSA level and the mean prostate volumes. In conclusion, the study states that fascin is secreted in healthy male controls and the levels did not differ according to age.

Fascin is a 55 kDa globular protein with an important role in cell migration. It is a member of a specific family of actin-bundling proteins [1]. The expression of fascin is greatly increased in many transformed cells, as well as in specialized normal cells including neuronal cells and antigen-presenting dendritic cells. The serum level of Fascin-1 is low or almost absent in the normal epithelia, but high levels of Fascin-1 were identified in various cancer types, with an important role in tumor progression, invasion and metastasis [2,3].

Overexpression of fascin immune-expression and its correlation with progressive high-grade tumors, great metastatic potential and poor prognosis had been found in several types of human neoplasms [4–8].

The aim of our study was to see if fascin is secreted in serum and to compare serum levels according to age in healthy male patients.

## Materials & methods

From 1 July 2016 to 31 December 2016, we collected serum from 46 males that were included in the prostate cancer screening cohort at the Urology clinic. Ethical approval was obtained from the Ethical Committee of the University of Medicine and Pharmacy, Tîrgu Mures, Romania (no. 52 from 24 March 2016). Each subject included in the study signed an informed consent and agreed to enter the study.

The collected serum was stored at -80°C at the Center for Advanced Medical and Pharmaceutical Research of the University of Medicine and Pharmacy Tîrgu Mures until serological determination was performed. We used to determine serum fascin level a quantitative sandwich ELISA kit from USBiological (Salem, MA, USA; Human fascin no.cat.024943, detection range 0.312–20 ng/ml), according to the manufacturer's standard protocol.

Briefly, 100 μl of serum, standards and blank were added to a precoated 96-well microplate at 37°C for 1 h. After discarding the reaction solution, a subsequent incubation was performed with appropriate detection of antibodies at 37°C for 1 h (100 μl). Following three washes and aspirations with washing solution, secondary antibodies (100 μl) were added to each well followed by incubation at 37°C for 30 min. After five additional aspirations and washes, a 90 μl of 3,3,5,5-tetramethylbenzidine solution was added into each well and incubated at 37°C for 25 min under light-protected conditions. Finally, a 50 μl of stop solution (2 M sulfuric acid) was added to stop the reaction, and the absorbance was measured immediately at 450 nm using a microplate reader (DSX™ Automated ELISA System Dynex Technologies, VA, USA).

### Statistical analysis

Statistical analysis was carried out using GraphPad InStat 3 software program. The Kolmogorov–Smirnov test was used to assess the normal distribution of continuous numerical variables. The results were presented as numbers and percentages for qualitative variables and as mean ± standard deviation or median values for quantitative variables. Means were compared using Fisher's Exact test. A value of p < 0.05 was considered statistically significant.

## Results

The included patients were divided into three subgroups: first group with age between 51 and 60 years old patients (11 patients), the second group included patients with age between 61 and 70 years (25 patients) and the third group contained patients with age between 71 and 80 years (ten patients).

Prostate-specific antigen (PSA) level was determined for each patient followed by prostate volume measurement and digital rectal examination. Due to clinical suspicion of prostate cancer (PCa) eight subjects underwent 12 core ultrasound guided biopsy. All eight patients had negative biopsies for PCa. In this subgroup mean serum fascin was 9.73 ng/ml (range: 7.35–13.65 ng/ml) and mean PSA: 9.05 ng/ml (range: 3.61–15.27 ng/ml).

The median age was 64 years (interquartile range [IQR]: 53–78). Mean fascin serum level was 9.84 ng/ml (range 3.96–17.63 ng/ml) and the mean PSA level was 2.74 ng/ml (range: 0.14–15.27 ng/ml). The mean prostate volume was 37.64 cc (range: 10–120 cc).

**Figure 1.**
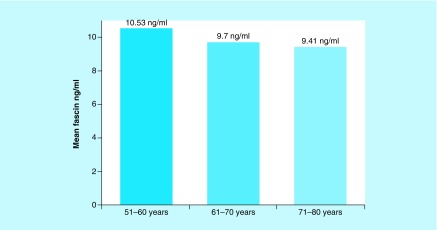
**Fascin serum levels in subgroups.**

The 51–60 years group had an mean fascin level of 10.53 ng/ml (range: 3.96–17.63 ng/ml), the 61–70 group a mean of 9.7 ng/ml (range: 4.92–14.36 ng/ml) and the 71–80 group had a mean of 9.41 ng/ml fascin serum level (range: 4.82–14.27 ng/ml), with no statistical difference of fascin serum level between groups (p = 0.65, Figure 1).

## Discussion

Fascin represents a highly studied protein in the current literature, due to its role in cancer progression and metastasis. The epithelial expression of fascin is clearly elevated in localized and hormone refractory PCa in comparison with healthy males, presenting benign prostate hyperplasia [6]. Fascin is an F-actin-bundling protein shown to stabilize filopodia and regulate adhesion dynamics in migrating cells [9,10], and its expression is correlated with poor prognosis and increased metastatic potential in a number of cancers [11].

Serum fascin determination was performed only on patients with small cell lung cancer [12], laryngeal carcinoma [13] and in patients with hepatocelular carcinoma (HCC) [14]. Serum level of fascin was associated with tumor aggressiveness in lung and laryngeal carcinomas, but still their results are presenting heterogeneous data regarding serum fascin in healthy individuals.

Our results are in concordance with Elewa *et al*. [14] findings about serum fascin in controls. They analyzed serum fascin using an ELISA kit from New East Biosciences in only 15 controls and the mean serum level of fascin was around 9.9 ng/ml (with 33.17 less than in HCC patients), value close to ours 9.84 ng/ml obtained after testing fascin in 46 males. Even more they found that in 50 patients with HCC, mean serum fascin was 14.8 ng/ml; significantly higher than in controls or cirrhotic patients (20 patients).

Further analysis of fascin levels is needed in order to establish the correlation between elevated fascin serum levels and biopsy proven PCa or in associations with other tissue and serum markers [15,16]. Furthermore, the sensitivity and the specificity of the determination can be evaluated. Including other parameters of the patients could improve our results, such as free-PSA and testosterone levels.

In 2015, Loeb *et al*. published an article about Prostate Health Index proving the importance in a multivariable approach reducing prostate biopsies and avoiding overtreatment [17]. Fascin determination in association with prostate health index could play a significant role in the diagnosis of clinically significant PCa.

Limitations of this study include those inherit to a pilot study. Although a larger number of subjects might increase the statistical strength of the study, still we report the largest cohort of males in which serum fascin was determined and we confirmed that fascin is secreted in serum of healthy males (without malignancies). As fascin might play a role as serum biomarker in metastatic cancer and could be a target for therapy, it is important to have a reference level to report at when investigating serum levels of fascin in cancer patients.

## Conclusion

Fascin is secreted in male's serum. From our data, serum level did not differ according to age in males and had a mean value of 9.84 ng/ml.

## Future perspective

Fascin was found to be secreted in serum of males according to our results. This might open further researches to identify if fascin could be a new biomarker for patients risk stratification or for predicting oncological outcomes in patients with cancer. As it is established that fascin plays a role in cell migration and metastasis, it is important to investigate fascin in serum of patients with neoplasia.

**Executive summary**

- Fascin is a 55 kDa globular protein with important role in cell migration.
- The expression of fascin is greatly increased in many transformed cells, as well as in specialized normal cells including neuronal cells and antigen-presenting dendritic cells.
- The serum level of Fascin-1 is low or almost absent in the normal epithelia, but high levels of Fascin-1 were identified in various cancer types, with important role in tumor progression, invasion and metastasis.
- Fascin serum levels could represent an accessible biomarker in prostate cancer.

**Study hypothesis**

- Fascin serum levels could differ in various age groups.
- We aimed to determine if there is a correlation between prostate-specific antigen and fascin serum levels.

**Discussion**

- Fascin is an F-actin bundling protein shown to stabilize filopodia and regulate adhesion dynamics in migrating cells, and its expression is correlated with poor prognosis and increased metastatic potential in a number of cancers.
- Serum fascin determination was performed only on patients with small cell lung cancer, laryngeal carcinoma and in patients with hepatocelular carcinoma.

**Conclusion**

- Fascin is indeed secreted in serum of healthy males with no statistical significance between age groups.
- This results could represent a stable reference point when measuring fascin serum levels in men.
